# Thymic hyperplasia in patients with acromegaly: imaging-based assessment and clinical implications

**DOI:** 10.20945/2359-4292-2026-0122

**Published:** 2026-10-11

**Authors:** Nur Yazdali Koylu, Hasbi Basaran, Busra Firlatan Yazgan, Suleyman Nahit Sendur, Selcuk Dagdelen, Macit Orhan Ariyurek, Tomris Erbas

**Affiliations:** 1 Department of Internal Medicine, Hacettepe University School of Medicine, Ankara, Türkiye; 2 Department of Radiology, Hacettepe University School of Medicine, Ankara, Türkiye; 3 Department of Endocrinology and Metabolism, Hacettepe University School of Medicine, Ankara, Türkiye

**Keywords:** Acromegaly, pituitary adenoma, thymus, thymic endocrinology, growth hormone

## Abstract

**Objective:**

We aimed to compare thymus visibility and volume in patients with acromegaly to those of controls, and to evaluate the correlation between growth hormone (GH), insulin-like growth factor-1 (IGF-1) levels, and thymus volume.

**Subjects and methods:**

This cross-sectional case-control study included 66 patients with acromegaly who underwent thoracic computed tomography scans. An age- and sex-matched control group of 65 trauma patients without comorbidities was selected. All thoracic computed tomography scans were reassessed to measure thymus volume using imaging software. Comparisons of thymus volume and assessment of correlation with GH and IGF-1 levels were performed.

**Results:**

The acromegaly group (28F/38M) and the control group (27F/38M) had similar sex and age distributions. The thymus was more frequently detectable in the acromegaly group (80.3%) compared to controls (44.6%; p = 0.001). Thymus volume was significantly higher in the acromegaly group (13.1 cm^3^ [9.7-18.7]) than in the control (10.3 cm^3^ [4.6-12.3]; p = 0.013). Serum IGF-1 levels were nearly significantly higher in acromegalic patients with a visible thymus compared to those without (visible thymus: 608.5 ng/mL [377.6-921.8]; non-visible thymus: 440.5 ng/mL [201.5-596]; p = 0.054). However, no significant differences were found in GH and IGF-1 upper limit of normal levels. No correlation was found between thymus volume and other parameters.

**Conclusion:**

Patients with acromegaly have larger and more frequently visible thymus glands than controls. When a mass is detected in the anterior mediastinum, acromegaly should be considered, especially before invasive procedures.

## INTRODUCTION

Acromegaly is a progressive disease characterized by excessive secretion of growth hormone (GH) and insulin-like growth factor 1 (IGF-1), most commonly due to a GH-secreting pituitary adenoma (^1^). It is a rare disorder, with a reported prevalence ranging from 2.8 to 13.7 per 100.000 individuals and annual incidence rates between 0.2 and 1.1 per 100.000 new cases (^2^). Patients typically present with enlargement of the extremities, coarsening of facial features, and soft tissue hypertrophy, and may develop various cardiovascular, metabolic, respiratory, gastrointestinal, and skeletal complications (^1^,^3^,^4^,^5^).

The thymus gland is a major target organ for GH and IGF-1 (^6^). GH receptors have been identified on thymic cells (^7^), and stimulation with GH and IGF-1 induces morphological and functional changes in thymic epithelial cells (TECs) and thymocytes (^6^,^8^,^9^). In addition, has been shown to GH promote thymocyte proliferation, growth, and differentiation (^7^).

To date, no case series has investigated changes in thymus volume in patients with acromegaly. We hypothesize that the thymus gland may increase in size and become radiographically detectable due to elevated serum GH and IGF-1 levels in these patients. The primary aim of this study was to compare thymus gland volume between patients with acromegaly and healthy individuals. The secondary aim was to investigate possible correlations between GH and/or IGF-1 levels and thymus gland volume.

## SUBJECTS AND METHODS

### Study design and study population

This single-center retrospective study was conducted at the Department of Endocrinology and Metabolism at a tertiary care university hospital. Medical records of patients diagnosed with acromegaly were retrospectively screened to identify those who had undergone thoracic computed tomography (CT) scans for any reason before or after transsphenoidal and/or transcranial surgery. Thoracic CT examinations were performed as part of routine clinical care for various indications, including assessment of comorbidities and suspected benign or malignant thoracic pathology. Patients who had thoracic CT performed during periods of elevated GH and IGF-1 levels, regardless of whether they had undergone pituitary adenoma surgery, were included. Patients with post-operative thoracic CT were included if hormonal remission had not been achieved, regardless of the time elapsed since surgery. An age- and sex-matched control group was selected from trauma patients without known disease who had undergone thoracic CT in the emergency department. This study was approved by the Ethics Committee of Hacettepe University (project ID: GO 22/497) and conducted in accordance with the Declaration of Helsinki.

### Study variables

#### Demographic, clinical, and anthropometric parameters

For both patient and control groups, age, sex, thymic appearance on thoracic CT (visible or not), and thymus volume (if visible) were recorded. In the patient group, additional data included height, weight, body mass index (BMI), pituitary adenoma size at diagnosis, history of surgery for acromegaly, and previous or current medical treatments (dopamine agonists, somatostatin receptor ligands [SRLs], pegvisomant). Disease duration was defined as the interval between the onset of acromegaly-related symptoms documented in medical records and the date of biochemical diagnosis. Comorbidities such as diabetes mellitus and hypertension were identified according to international guidelines (^10^,^11^). Height and weight were not available for the control group, as these measurements are not routinely recorded in emergency settings. To compare body fat distribution between groups, single-slice subcutaneous adipose tissue area (cm^2^) was measured at the T_12_ vertebral level on thoracic CT (^12^,^13^). Measurements were performed using syngo.via software (Siemens Healthineers, Germany). A Hounsfield unit (HU) range of -200 to -50 HU was used for the assessment of subcutaneous adipose tissue area. The BMI was calculated as weight (kg) divided by height squared (m^2^).

#### Radiologic evaluation of thymus gland

All thoracic CT scans were performed at the same tertiary center and re-assessed and reported in consensus by two experienced radiologists who were blinded to the clinical data, to identify the thymus gland and calculate its volume (if visible). Thymus gland volume was measured with imaging software (Syngo. via, Siemens Healthineers). For visible glands, tissue boundaries were defined, and density between -200 and +50 HU was used for delineation. Measurements were not performed in patients whose thymus was not visualized. The proportions of visible thymuses and their volumes were compared between groups.

#### Assessment of serum GH and IGF-1 levels

Serum GH and IGF-1 levels corresponding to the date of the thoracic CT were retrieved from the electronic medical records. Serum samples were collected in the early morning after a 12-hour overnight fasting period. Hormonal remission was defined as normalization of age-adjusted serum IGF-1 levels (less than the upper limit of normal [ULN]), random serum GH <1 µg/L, or a nadir <0.4 µg/L after a 75 g oral glucose load (^14^,^15^). The ULN was determined according to the assay reference for IGF-1, and age-adjusted IGF-1 was defined as the patient's IGF-1 divided by the ULN.

### Statistical analysis

Statistical analysis was performed with IBM SPSS Statistics v.26.0. Normality was assessed using visual methods (histograms and probability plots) and the Kolmogorov-Smirnov test. Continuous variables were summarized as mean ± standard deviation for normally distributed data, or as median and interquartile range (IQR) for non-normally distributed data. Categorical variables were presented as frequency and percentage. For group comparisons, Pearson's Chi-square test was used for categorical variables, and the Mann-Whitney U test for non-normally distributed continuous variables. A two-tailed p-value of <0.05 was considered statistically significant.

## RESULTS

### Demographic and clinical characteristics of patients with acromegaly and healthy controls

A total of 397 patients diagnosed with acromegaly were screened, and *66* (28 F/38 M) with thoracic CT scans were included. The control group comprised 65 individuals (27 F/38 M) who underwent thoracic CT for trauma. The mean (± standard deviation) age at thoracic imaging was 49.5 ± 13.8 years in the acromegaly group versus 51.4 ± 14.1 years in the controls (p = *0.48;* **Table 1).** No significant difference was observed in the median (IQR) subcutaneous adipose tissue area between groups (acromegaly: 94.3 cm^2^ [40.8-145.2]; control: 85.7 cm^2^ [54.9-155.4]; p = 0.80). The median (IQR) BMI in the acromegaly group was 31.4 kg/m^2^ (27.1-33.6).

**Table 1:** Demographic, clinical, and biochemical characteristics of patients with acromegaly

**Variable**	**Acromegaly group (n = 66)**
Demographic data	
Female, n (%)	28(41.8)
Age, years^a^	49.5 ±13.8
Duration of disease, years^b^	7.5(4.3-9)
Height, cm^a^	171.3 (±13.5)
Weight, kg^b^	89.5(80-106)
BMI, kg/m^2^ kg/m^2b^	31.4(27.1-33.6)
GH, ng/mL^b,c^	6(2.4-14.8)
IGF-1, ng/mL^b,c^	594(317-887.5)
IGF-1, xULN^a,c^	1.53(1.03-2.43)
Adenoma characteristics^d^	
Macroadenoma, n (%)	43(79.6)
Adenoma size, mm^b^	17.5(9.3-22)
Adenoma size (min-max), mm	4-45
Surgical intervention, n (%)	62 (94)
Medical treatment, n (%)^e^	45 (68)
Dopamine agonist, n (%)	10(^15^)
Pegvisomant, n (%)	5(7.5)
Somatostatin receptor ligands, n (%)	41 (62)
Radiotherapy, n (%)	9(^14^)
Comorbities	
Coronary artery disease, n (%)	4(^6^)
Diabetes mellitus, n (%)	23 (38)
Hypertension, n (%)	28 (46)
Hypopituitarism, n (%)	19(^30^)

BMI: body mass index; GH: growth hormone; IGF-1: insulin-like growth factor-1; ULN: upper limit of normal. ^a^Mean (± standard deviation); ^b^median (interquartile range); ^c^serum levels of acromegaly patients who had chest computed tomography; ^d^missing data; ^e^three patients received all three treatment modalities, three patients received dopamine agonists and somatostatin receptor ligands, and two patients received both pegvisomant and somatostatin receptor ligands.

The median (IQR) disease duration in acromegaly was 7.5 (4.3-9) years. The median pituitary adenoma size was 17.5 mm (IQR: 9.3-22; min-max: 4-45); mac-roadenoma (>1 cm) was detected in 43 (79.6%) patients. The median (IQR) serum GH and IGF-1 levels were 6 ng/mL (2.4-14.8) and 594 ng/mL (317-887.5), respectively, and IGF-1 xULN was 1.53 (1.03-2.43). No statistically significant correlation was identified between thymus volume and BMI, adenoma size, GH, IGF-1, or IGF-1 xULN **(Table 2).** Most patients (91%) underwent transsphenoidal surgery. Craniotomy was performed in two (3%) patients and four (6%) underwent no surgery. Nine (14.1%) patients received radiotherapy. Histopathological confirmation of pituitary adenoma was available in most operated patients; however, pathology reports were unavailable in 10 (16%) patients.

**Table 2:** Association between thymus volume and clinical parameters

**Parameters**	**Thymus gland volume**	
	***r***	*P*
Age, years	0.16	0.30
BMI, kg/m^2^	0.16	0.40
Adenoma size, mm	0.05	0.80
GH, ng/mL	0.05	0.77
IGF-1, ng/mL	0.17	0.30
IGF-1,xULN	0.27	0.08

BMI: body mass index; GH: growth hormone; IGF-1: insulin-like growth factor-1; ULN: upper limit of normal.

The SRLs were the most common medical treatment (62%). Thirty-one patients (47%) received SRLs as monotherapy; two (3%) were treated with dopamine agonists. Three (5%) received all three treatment modalities, five (7.5%) received dopamine agonists and SRLs, and two (3%) received both pegvisomant and SRLs. The most prevalent comorbidities among acromegaly patients were hypertension (46%) and diabetes mellitus (38%). Hypopituitarism was present in 30% of the patients.

### Thymus volume and appearance in acromegaly versus control

In the acromegaly group, 53 patients (80.3%) had a visible thymus on thoracic, CT compared with 29 (44.6%) in the control group (p = 0.001). Thymus volume was significantly greater in the acromegaly group (13.1 cm^3^ [9.7-18.7]) than the control (10.3 cm^3^ [4.6-12.3]; *p* = 0.013; **Table 3**). Age and subcutaneous adipose tissue area did not significantly differ between patient and control groups when stratified by thymus visibility. No sex differences were observed in either group among those with or without a visible thymus.

**Table 3:** Thymus gland volumes and visibility in acromegaly and control groups

**Variable**		**Groups**		**Acromegaly vs. control.** *P*
		**Acromegaly** (n = **66)**	Control (n = 65)	
Sex, n (F/M)	Visible thymus	53(21/32)	29(10/19)	0.72
	Not visible	13(7/6)	36(17/19)	0.68
	*P*	0.39	0.30	
Age, years	Visible thymus	45.5 (36-55.3)^b^	46.1 (±14.7)^a^	0.73
	Not visible	63(55-71)	55.7 (±12.2)	0.11
	*P*	**0.001**	**0.006**	
BMI,kg/m^2b^	Visible thymus	31.1 (27.3-41.2)	—	
	Not visible	32.6(26.2-33.7)	—	
	*P*	0.73	—	
Subcutaneous adipose tissue area, cm^2b^	Visible thymus	79.8(37.5-141.5)	61.3(35.5-110.8)	0.41
	Not visible	131.3(94.2-183.3)	102(76-175.1)	0.24
	*P*	**0.02**	**0.03**	
Disease duration, years	Visible thymus	8(4-13)	—	
	Not visible	12(8-15)	—	
	*P*	0.1	—	
Adenoma size, mm^bc^	Visible thymus	17.5(10-24.5)	—	
	Not visible	18.5(10.3-21.6)	—	
	*P*	0.93	—	
Thymus visibility, n(%)	Visible thymus	53(80.3)	29(44.6)	**0.001**
	Not visible	13(19.4)	36(55.4)	
Thymus volume, cm^3 b^		13.1 (9.7-18.7)	10.3(4.6-12.3)	**0.013**

BMI: body mass index; F: female; GH: growth hormone; IGF-1: insulin-like growth factor-1; M: male. ^a^Mean (± standard deviation); ^b^median (interquartile range); ^c^missing data.

### Characteristics of the acromegaly group based on thymus visibility

In the acromegaly group, the thymus was visible in 53 (80.3%) patients and not visible in 13 (19.4%). There was no significant difference in sex distribution between the groups with visible (21 F/32 M) and non-visible (7 F/6 M) thymus. Patients without visible thymus were significantly older than those with visible thymus (visible: 45.5 years [36-55.3]; non-visible: 63 years [55-71]; p = 0.001). Although BMI values were similar between acromegaly patients with and without visible thymus glands, subcutaneous adipose tissue area was higher in patients without visible thymus glands. Disease duration did not significantly differ between those with visible and non-visible thymus (visible: 8 years [4-13]; non-visible: 12 years [8-15]; p = 0.1).

No significant difference in pituitary adenoma size was found between those with visible and non-visible thymus glands. Serum IGF-1 levels were nearly significantly higher in patients with a visible thymus compared to those without (visible: 608.5 ng/mL [377.6-921.8]; non-visible: 440.5 ng/mL [201.5-596]; *p* = 0.054). Nevertheless, there was no statistically significant difference in GH levels (visible: 7.9 ng/mL [2.1-18.8]; non-visible: 2.8 ng/mL [1.5-6.4]; p = 0.09) or IGF-1 x ULN (visible: 1.6 [1-2.5]; non-visible: 1.5 [0.8-4.3]; p = 0.95).

Among the *66* patients with acromegaly, 26 had thoracic CT during the active preoperative phase, whereas 29 had CT after pituitary surgery. Notably, 11 had both pre- and post-operative thoracic CT Of the 26 patients with acromegaly who underwent thoracic CT only preoperatively, 19 had a visible thymus, and 25 of 29 with post-operative CT had a visible thymus. The median (IQR) interval between preoperative CT and surgery was two months (1-5); for post-operative CT, it was nine months (4-35.5). Thymus volume did not differ significantly between groups (preoperative: 13 cm^3^ [9.7-19]; post-operative: 12.4 cm^3^ [7.2-16.8]; p = 0.61). Furthermore, GH levels were higher in preoperative scans (preoperative: 7.2 ng/mL [3.3-25.8]; post-operative: 3.9 ng/mL [1.4-10.9]; p = 0.01). There was a nearly significant difference in IGF-1 levels (preoperative: 615 ng/mL [435-892]; post-operative: 442 ng/mL [202.8-851.3]; p = 0.053), although IGF-1 x ULN was significantly higher in the preoperative group (preoperative: 2 [1.3-2.7]; post-operative: 1.3 [0.6-1.9]; p = 0.002).

Among 11 patients with both pre- and post-operative thoracic CT, thymus volume was measurable at both points in seven. In the remaining four, thymus volume could not be measured preoperatively in two, or post-operatively in two. The median (IQR) duration between pre- and post-operative CT was 43.5 months (24-104). After surgery, hormonal remission was achieved in five patients, and four of those had a decrease in thymus volume **(Table 4).** A volume increase was observed in two patients who did not achieve hormonal remission **(Figure 1)**.

**Table 4:** Characteristics of patients with both pre- and post-operative thorax computed tomography scans

**Case**	**Sex**	**Age (years)**	**BMI (kg/m^2^)**	**IGF-1/ULN**		**Presence of residual .or recurrent disease . and treatment**	**Thymus volume (cm^3^)**		**Volume difference**	**CT-surgery interval (mo)**		**Interval between bothCTs (mo)**	**Clinical status**
				**Pre-op**	**Post-op**		**Pre-op**	**Post-op**		**Pre-op**	**Post-op**		
1	M	46	27.49	3.04	0.75	Residual, IMRT+, SRLS +	15.26	17.23	**↑**	1	106	107	Rem
2	M	56	-	2.82	1.66	NED, no ST/RT	22.66	15.88	***↓***	2	1	**3**	Rem
3	M	55	32.7	2.58	1.26	Residual, no RT, SRLs +	12.45	15.42	**↑**	23	68	**91**	Per
4	M	58	30	1.79	1.69	NED, no RT, DA + SRLs	19	11.80	***↓***	1	48	**49**	Rem
5	F	43	33.60	1.35	0.36	Residual, no RT, DA +	17.78	12.07	***↓***	5	172	177	Rem
6	M	62	30.44	1.99	3.10	Residual, no RT, DA + SRLs + P	6.01	19.41	**↑**	1	106	107	Per
7	M	37	22.4	1.90	0.74	Residual, no RT, SRLs +	13.48	7.15	***↓***	2	21	**23**	Rem

BMI: body mass index; CT computed tomography; DA: dopamine agonist; IMRT: intensity-modulated radiation therapy; IGF-1: insulin-like growth factor 1; mo: months; NED: no evidence of disease; P: pegvisomant; Per: persistant; Rem: remission; RT: radiotherapy; SRL: somatostatin receptor ligand; ST: systemic therapy; ULN: upper limit of normal. Within the acromegaly group, seven patients had visible thymus glands on both pre- and post-operative chest CT scans. Following surgery, hormonal remission was achieved in five patients, and thymus gland volume decreased in four of them. Conversely, an increase in thymus gland volume was observed in two patients who did not achieve hormonal remission.

**Figure 1:**
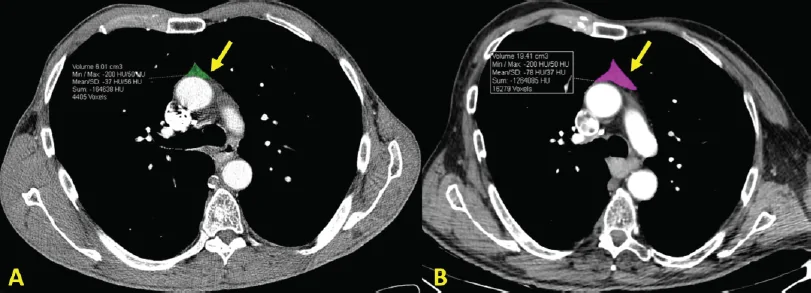
Enlargement of the thymus gland in Case 6, a patient with acromegaly who exhibited persistent disease despite transsphenoidal surgery and medical treatment. (**A**) Preoperative thymus volume: 6.01 cm^3^; insulin-like growth factor-1/upper limit of normal: 1.99. (**B**) Postoperative thymus volume: 19.41 cm^3^; insulin-like growth factor-1/upper limit of normal: 3.10.

Thymus volume in the acromegaly group was additionally analyzed according to SRL therapy. There were no significant differences in thymus volume between patients treated with SRLs (14.64 cm^3^ [± 7.03]) and those not treated (14.75 cm^3^ [± 9.22]; *p = 0.96)*.

### Characteristics of the control group based on thymus visibility

In the control group, the thymus was visible in 29 (44.6%) and not visible in 36 (55.4%). No significant difference in sex distribution was found between those with a visible thymus (10 F/19 M) and those without (17 F/19 M) (*p = 0.30)*. Controls without a visible thymus were older than those with a visible thymus (visible: 46.1 years [± 14.7]; non-visible: 55.7 years [± 12.2]; p = 0.006). Subcutaneous adipose tissue area was greater in controls without a visible thymus (visible: 61.3 cm^2^ [35.5-110.8]; non-visible: 102 [76-75.1]; *p = 0.03*). No comorbidities were present in the control group.

## DISCUSSION

Our study demonstrated that the thymus gland was more frequently visible and larger on thoracic CT scans in patients with acromegaly than healthy individuals. To the best of our knowledge, this is the first case series to systematically evaluate thymic enlargement in acromegaly. Thymus gland volume may vary depending on whether hormonal remission is achieved, as indicated by the comparison of preoperative and post-operative thoracic CT scans.

The association between pituitary hormones and thymic tissue has been recognized since early hy-pophysectomy experiments, which demonstrated thymic involution after anterior pituitary ablation (^16^). Subsequent research established the concept of bidirectional thymic endocrinology, highlighting reciprocal interactions between pituitary hormones and thymic function (^17^).

Thymocytes and TECs express GH receptors, identifying the thymus as a target organ for GH (^6^). Accordingly, in patients with acromegaly, who exhibit abnormally high GH synthesis, enlargement of the thymus is to be expected. In 1990, Bazzoni and cols. (^18^) reported a 23-year-old female patient with uncontrolled acromegaly who underwent a thoracic CT three years after diagnosis and identified a homogeneous mass in the mediastinum. More recently, a similar presentation was reported in a male patient whose thymic mass regressed following the surgical treatment of a pituitary adenoma (^19^). In our study, patients with acromegaly had a more frequently visible and larger thymus gland compared with healthy individuals, which is consistent with previous case reports. Timsit and cols. (^20^) reported a strong correlation between serum thymulin and IGF-1 levels in acromegaly patients, suggesting a potential link between circulating IGF-1 and thymus size. However, no significant correlation was observed between thymus volume and GH, IGF-1, or IGF-1 xULN in the overall acromegaly cohort. This finding suggests that thymic enlargement may reflect cumulative hormonal exposure rather than hormone levels measured at a single time point.

Human and animal studies have suggested that locally produced GH by thymic cells, as well as the expression of GH receptors in TECs and certain thymocytes, may contribute to increased thymus gland volume through paracrine effects (^21^,^22^,^23^). In addition to GH receptors, somatostatin receptors have also been identified on the thymus (^24^,^25^,^26^). Transcripts of SST_1_ SST_2A_, and SST_3_ somatostatin receptor subtypes have been detected in human thymic tissue (^24^,^25^). As somatostatin, believed to play a role in intrathymic regulation, is administered as an analogue in the treatment of acromegaly, it may influence thymus gland volume. *In vitro* studies have demonstrated that somatostatin and its analogue, octreotide, elicit inhibitory responses on TECs (^24^). The clinical use of SRLs in thymic tumors and the role of SST scintigraphy support these experimental findings regarding thymic regulation (^27^,^28^,^29^). In our study, evaluation of SRL treatment effects on thymus volume revealed no significant difference, which may be attributed to the small sample size.

To date, no case series has investigated the potential link between acromegaly and thymus gland enlargement. To our knowledge, this is the first study to demonstrate a relationship between acromegaly and thymic hyperplasia. Nevertheless, our study has limitations. The retrospective design inherently limits the reliability of data collection. In the acromegaly group, patients were not selected according to a predefined imaging protocol; rather, we retrospectively included individuals with biochemically active disease who had available thoracic CT imaging documented in their medical records. Because the indication for thoracic CT could not be reliably determined for all cases, we cannot fully exclude the possibility that CT availability or the clinical context may have influenced study selection. Although thoracic imaging is not routinely performed in all patients with acromegaly, it may be ordered for the assessment of comorbidities in certain clinical scenarios. Importantly, none of the included patients had a known thymic disorder or radiological findings suggestive of thymic pathology. In addition, although rare ectopic GHRH-secreting thymic neuroendocrine tumors have been described in the literature, no clinical, radiological, or pathological evidence of this diagnosis was found in our cohort. Therefore, these considerations should be taken into account when interpreting our findings.

Cumulative exposure to GH and IGF-1, as well as the duration of biochemical control, may be more relevant determinants of thymic size than single time-point measurements. However, due to the retrospective study design and incomplete longitudinal data, accurate assessment of cumulative hormonal burden was not feasible in our cohort.

Another limitation is that some thoracic CT examinations were contrast-enhanced, whereas others were not. Nonetheless, thymus volume measurements were performed using density-based segmentation with a predefined HU threshold range (-200 to +50 HU), which was applied consistently to all scans. Previous CT studies have demonstrated that normal thymic attenuation values remain within this range even after contrast administration, suggesting that contrast enhancement is unlikely to substantially compromise volumetric assessment (^30^).

Factors including age, sex, and BMI may influence thymus gland volume (^31^,^32^,^33^). While the patient and control groups were similar with respect to age and sex distribution, the absence of BMI data in the control group may have influenced group comparisons. To partially address this, subcutaneous adipose tissue area at the T12 vertebral level was measured using thoracic CT in both groups, based on previous studies demonstrating significant correlations between adipose tissue measurements at T12 and L3 vertebral levels (^12^,^13^). No significant difference in subcutaneous adipose tissue area was observed between the acromegaly and control groups. Furthermore, when the patient and control groups were evaluated separately, increased subcutaneous adipose tissue area was associated with reduced thymus visibility, consistent with previous reports suggesting that adiposity contributes to thymic fatty involution (^31^).

Normal aging-related changes may complicate radiological assessment of the thymus gland. The thymus involutes after puberty, being largest in infants (^34^). It is generally expected to become indistinguishable on cross-sectional imaging during the third and early fourth decades of life (^31^,^35^). Increased fat infiltration with age decreases thymic attenuation from 80 HU to 56 HU on CT, thereby reducing thymus visibility (^36^). Although there was no significant age difference between patient and control groups, individuals with nonvisible thymus glands were older than those with visible thymus glands in both groups, suggesting a potential influence of age on thymus gland volume. Two separate studies involving individuals aged 20-30 and 40-69 years indicated that changes in thymic fat were less pronounced in women, with higher HU values observed (^32^,^33^). This results in increased thymus visibility in women. Nonetheless, we did not find any significant difference in thymus gland visibility between sexes in either the acromegaly or healthy control groups, a discrepancy possibly attributable to the smaller sample sizes compared to those in prior studies.

In conclusion, patients with acromegaly exhibit larger and more frequently visible thymus glands compared to healthy controls. When an anterior mediastinal mass is detected on thoracic imaging, acromegaly should be considered in the differential diagnosis before invasive procedures are planned.

## Data Availability

datasets related to this article will be available upon request to the corresponding author.
